# Restaurant and Bar Owners’ Exposure to Secondhand Smoke and Attitudes Regarding Smoking Bans in Five Chinese Cities

**DOI:** 10.3390/ijerph8051520

**Published:** 2011-05-12

**Authors:** Ruiling Liu, S. Katharine Hammond, Andrew Hyland, Mark J. Travers, Yan Yang, Yi Nan, Guoze Feng, Qiang Li, Yuan Jiang

**Affiliations:** 1 Chinese Center for Disease Control and Prevention, 27 Nanwei Rd, Beijing 100050, China; E-Mails: yangyan8288@hotmail.com (Y.Y.); haversian@sohu.com (Y.N.); fengguoze@hotmail.com (G.F.); jiangyuan88@vip.sina.com (Y.J.); 2 School of Public Health, University of California Berkeley, 50 University Hall #7360, Berkeley, CA 94720, USA; E-Mail: hammondk@berkeley.edu; 3 Department of Health Behavior, Roswell Park Cancer Institute, Elm & Carlton Streets, Buffalo, NY 14263, USA; E-Mails: andrew.hyland@roswellpark.org (A.H.); mark.travers@roswellpark.org (M.J.T); 4 Department of Psychology, University of Waterloo, 200 University Avenue West, Waterloo, ON, N2L 3G1, Canada; E-Mail: qangli33@yahoo.com

**Keywords:** knowledge, attitudes, secondhand smoke, smoking ban, restaurant and bar

## Abstract

Despite the great progress made towards smoke-free environments, only 9% of countries worldwide mandate smoke-free restaurants and bars. Smoking was generally not regulated in restaurants and bars in China before 2008. This study was designed to examine the public attitudes towards banning smoking in these places in China. A convenience sample of 814 restaurants and bars was selected in five Chinese cities and all owners of these venues were interviewed in person by questionnaire in 2007. Eighty six percent of current nonsmoking subjects had at least one-day exposure to secondhand smoke (SHS) at work in the past week. Only 51% of subjects knew SHS could cause heart disease. Only 17% and 11% of subjects supported prohibiting smoking completely in restaurants and in bars, respectively, while their support for restricting smoking to designated areas was much higher. Fifty three percent of subjects were willing to prohibit or restrict smoking in their own venues. Of those unwilling to do so, 82% thought smoking bans would reduce revenue, and 63% thought indoor air quality depended on ventilation rather than smoking bans. These results showed that there was support for smoking bans among restaurant or bar owners in China despite some knowledge gaps. To facilitate smoking bans in restaurants and bars, it is important to promote health education on specific hazards of SHS, provide country-specific evidence on smoking bans and hospitality revenues, and disseminate information that restricting smoking and ventilation alone cannot eliminate SHS hazards.

## 1. Introduction

Secondhand smoke (SHS) has been proven to cause several diseases affecting many organs in human beings. The adverse health effects of SHS and the benefits of smoking bans have been well documented [1–7]. Restaurants and bars are very important public places for many people, and they are also workplaces for restaurant or bar workers. As of January 2011, 55 nations have enacted 100% smoke-free laws, with 37 including both restaurants and bars [8]. Despite progress, only 9% of countries mandate smoke-free bars and restaurants [9].

In mainland China in 2010, 26% of its urban adult population and 30% of its rural adult population were current smokers, with about 70% of its adult population exposed to SHS in a typical week [10]. Smoking is nationally prohibited in the following public places: gymnasiums; libraries, museums and art galleries; waiting rooms for public transportation; passenger trains, passenger ships and flights; and classrooms, activity rooms and dorms for minors [11], and it was generally not regulated in restaurants and bars before 2008.

In 2005, China ratified the World Health Organization Framework Convention on Tobacco Control (WHO FCTC). Since then, there have been several initiatives to reduce exposure to SHS in public places, including restaurants and bars. In January 2007, the Beijing Health Bureau and the Chinese Center for Disease Control and Prevention (CDC) sent 40 thousand letters to restaurant and bar owners in Beijing asking them to voluntarily prohibit smoking (not allowing smoking at all in any dining areas) in their own establishments [12]. On May 1st, 2008, the Beijing government passed a law actually requiring restaurants within the city to prohibit or restrict smoking (only allowing smoking in designated sections of dining areas) [13]. Although several other large cities, including Shanghai, Guangzhou, Hangzhou, and Yinchuan, have taken steps to reduce SHS exposure by regulations, restrictions on smoking in restaurants and bars are mostly voluntary, as was true in Beijing before May 2008. There are still no regulations on smoking in bars across China.

As more and more tobacco control activities have been initiated in China, the public’s awareness of tobacco hazards has increased. Between 1996 and 2002, awareness of the health effects of smoking increased from 40% to 70% for lung cancer and from 4% to 21% for heart disease, and the percentage of the public who knew that passive smoking (exposure to SHS) could cause serious health effects increased from 15% to 30% [14].

According to a study conducted in seven Chinese cities in 2006, 29% of the nonsmoking residents and 20% of the smoking residents supported restricting smoking in restaurants and bars, and 42% of the nonsmoking residents and 24% of the smoking residents supported prohibiting smoking in these venues [12]. In January 2007, when the Beijing Health Bureau and the Chinese CDC called for voluntary smoking prohibition to thousands of restaurant owners, 86% of the respondents showed their support for prohibiting or restricting smoking in restaurants, but 52% of the respondents also expressed their concern about the potential negative influence on their revenue [12].

Supportive public attitudes are often necessary for facilitating the process of passing smoke-free legislations or regulations by local or national governments [15]. To examine the public attitudes towards banning smoking in restaurants and bars in China, a study was conducted by the Chinese CDC in 2007. It is hoped that the study results can convey key information to China and other countries which are working on creating smoke-free restaurants and bars.

## 2. Methods

Five cities were selected for the study with consideration of both locations and local health institutes’ interests in the study: Beijing (in northern China), Xi’an (in central western China), Wuhan (in central China) and Kunming and Guiyang (both in southwest China). The total population of the five cities was 36,390,000, 6.3% of the total urban population in China in the end of 2006 [16].

A total of 814 restaurants and bars were selected in the five cities using the method described previously [17]. First, one urban district and one suburban district were selected based on convenience sampling in each city. Next, different types of restaurants and bars were selected in proportion to the numbers of these venues listed in the city’s online yellow pages, resulting in approximately 50 Chinese dining, five Chinese fast food, five Western dining, five Western fast food restaurants and 15 bars in each district. Lastly, the research team from the local CDC or an equivalent health education institute in each city invited owners to participate in the study by phone if a customer service number was available or by visiting the venues in person and asking owners to participate in the study. All owners were told that the study was interested in aggregated results rather than individual data, and that all their information would be kept confidential. Oral consent was obtained from each participating owner. Questionnaire interviews of owners from all these venues were conducted and field measurements of a SHS tracer, fine particulates matters (PM_2.5_), were also conducted in 404 of these venues. This convenience sampling approach was used because of the budget limit as well as logistic issues for field survey and instrumental measurements. This paper only reports the results of questionnaire interviews of owners and results of the field measurements were reported elsewhere[17].

In-person interviews of restaurant or bar owners were conducted from July to September 2007 by trained personnel in each city with a standard protocol developed by the Chinese CDC. When an owner was not available during the study period (usually owners of large restaurants or bars) the venue manager was interviewed instead after he/she contacted with the owner by phone for authorization to participate in the survey.

A structured questionnaire was used for in-person interviews to obtain information. Subjects were asked about the smoking policies in their venues, that is, whether it prohibited (did not allow smoking at all in dining areas), restricted (allowed smoking in designated sections of dining areas only) or allowed smoking (allowed smoking everywhere in dining areas). Information on subjects’ demographic characteristics, current smoking status (defined by ever smoking during the month before interview) and exposure to SHS at work in the past week was also collected. Six questions were asked to examine subjects’ knowledge of the effects of tobacco smoke on health, specifically, whether smoking is harmful to smokers’ health; whether passive smoking is harmful to nonsmokers’ health; whether passive smoking can cause lung cancer; whether passive smoking can cause heart diseases; whether children with smoking parents are more likely to develop asthma or other respiratory diseases than those with nonsmoking parents; and whether women with smoking husbands are more likely to get lung cancer than those with nonsmoking husbands. Subjects were also asked about their views on smoking bans in public places, including restaurants and bars. Whenever possible, questions were adapted from a validated questionnaire used by an ongoing Chinese national project during the study period, *Monitoring Passive Smoking in Public Places in 2007*, which was conducted by the Chinese CDC and interviewed subjects from the general population from 32 provinces and municipalities [18]. New questions were also developed for this study to assess whether subjects believed that smoking bans would reduce hospitality revenues, whether they were willing to ban smoking in their own venues, and the reasons for their positions. This study was approved by the Ethics Review Board of the Chinese CDC.

All data were analyzed using Stata/IC 10.0 (College Station, TX, USA). Subjects’ exposure to SHS, their knowledge of tobacco smoke and health, their support for smoking bans in restaurants and bars, and the reasons for their unwillingness to ban smoking in their own venues were described. Backward stepwise logistic regression models were used to explore factors that would impact respondents’ attitudes towards smoking bans in restaurants or bars. Explanatory variables including city, type of venue, subjects’ age, gender, education, current smoking status, knowledge on tobacco smoke, and opinions on smoking bans and hospitality revenues. Subjects’ overall knowledge of health effects of tobacco smoke was graded in a 6 point scale, with one point given for each correct answer. Potential interactions effects between gender and smoking status, age and smoking status, smoking status and knowledge scores, and education level and knowledge scores were considered for model developments. Wald tests were used to test restricted versus full models, interaction items and variables with *p* values of Wald test greater than 0.20 were excluded from the model.

## 3. Results

A total of 814 establishments (665 restaurants and 149 bars) were included in the study, and all these venues’ owners or managers were interviewed. Fifty one percent of the subjects were males, 30% were more than 35 years old, 45% had higher than high school education, and 35% were current smokers (Table 1). Subjects from Wuhan were relatively order and subjects from Guiyang had relatively higher education than those in other cities. No significant differences in other demographic characteristics were observed between subjects from different cities.

### 3.1. Smoking Policy and Exposure to Secondhand Smoke

According to subjects’ reports, 64 (9.6%) restaurants and nine (6.0%) bars prohibited smoking, 221 (33%) restaurants and 35 (23%) bars restricted smoking. Eleven percent of current nonsmoking owners voluntarily prohibited smoking in their venues, while only 6% of current smoking owners did so. For current nonsmoking subjects, 49% of those from restaurants prohibiting smoking and all of those from bars prohibiting smoking reported exposure for at least one day at work during the past week (Table 2). Overall, 86% of nonsmoking subjects from restaurants and 90% of those from bars had at least one-day exposure to SHS at work in the past week.

### 3.2. Knowledge of Tobacco-Related Health Effects

**Figure 1.**
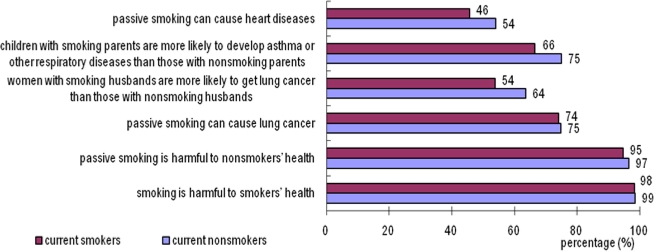
Restaurant and bar owner and manager awareness of smoking-related health effects, five Chinese cities, 2007. Note: results presented in this figure represent simple aggregation of the survey results, and no sampling weighting was used.

More than 95% of subjects believed that smoking or passive smoking was harmful to health, 75% knew that passive smoking could cause lung cancer, but only 61% believed that women with smoking husbands had higher risk to develop lung cancer. 72% believed that children with smoking parents were more likely to develop asthma or other respiratory disease and 51% knew that SHS could cause heart disease (Figure 1). Overall, 37% of the subjects answered all the six health-related questions correctly.

### 3.3. Attitudes Towards Smoking Bans in Hospitality Venues

Over 85% of subjects supported prohibiting smoking in schools and public vehicles, 73% in hospitals, and 58% in offices. However, only 17% supported prohibiting smoking in restaurants and 11% supported in bars, and their support for prohibiting smoking was much lower than their support for restricting smoking (Table 3).

As shown in Table 4, although 80% of subjects supported prohibiting or restricting smoking in restaurants and 62% supported this for bars, only 53% of the subjects were willing to do so in their own establishments in the absence of government regulations. Fifty five percent of subjects thought that prohibiting or restricting smoking would reduce hospitality revenues.

Table 5 presents the results of logistic regression analysis to explore factors related to supportive attitudes towards prohibiting or restricting smoking in restaurants and bars. Non-smoking subjects, those who knew more about tobacco smoke hazards, and those who agreed that prohibiting or restricting smoking would not reduce their revenues, were more likely to support prohibiting or restricting smoking in restaurants or bars, and more willing to do so in their own venues when other factors were adjusted. Comparing to subjects from Beijing, subjects from Wuhan were less likely to report their support, while subjects from Guiyang were similarly supportive or willing to prohibit or restrict smoking in their own venues. Subjects of bars and Chinese restaurants were less willing to do so in their own venues than those of western restaurants.

Of those who were unwilling to prohibit or restrict smoking in their own venues, 82% worried that it would reduce their revenues, 63% said that indoor air quality depended more on ventilation than smoking bans, and 62% said prohibiting or restricting smoking was not their restaurants’ or bars’ duty.

## 4. Discussion

As a part of the same project as this study, field observations of smoking policies were conducted in 404 of the 814 venues included in this study, which were reported elsewhere [17]. Field observations showed that only 6.9% (23/333) of restaurants and no bars actually prohibited smoking voluntarily and 2.1% (7/333) of restaurants and 2.8% (2/71) of bars actually restricted smoking voluntarily [17]. This indicates over-reporting of existing voluntary smoking prohibition or restriction by subjects in this study.

Together, the field observations reported previously and the results from the present study may provide the upper and lower bounds for existing voluntary smoking policies in restaurants and bars included in this study. Results of this study showed that exposure to SHS was very common even in restaurants and bars prohibiting smoking. One reason might be the misclassification of smoking policies introduced by potential over-reporting of smoking prohibition in restaurants and bars, as discussed above. Alternatively, this finding might be a result of poor compliance to voluntary smoking prohibition.

According to the *China Labor Statistical Yearbook 2006* [19], only 19% of the working population in Beijing had higher than high school education, which is lower than the education attainments of restaurant/bar owners or managers in this study. This may be a reason why subjects of this study had relatively higher awareness of the negative health effects caused by smoking or SHS than that reported in previous studies mentioned in the introduction part. Alternatively, the subjects might have over reported their education attainments.

Subjects’ knowledge of specific hazards of tobacco smoke was much more limited, for example, only half of the subjects knew that passive smoking can cause heart disease. Similar results were found in patrons recruited from the same venues [20]. This implies that health education related to tobacco smoke and warning labels on tobacco products in China should focus on specific health hazards rather than general, vague information. Up until October 2008, the health warning on cigarette packs in China had always been “smoking is harmful to your health”, and since October, 2008, a new health warning that “Quitting smoking reduces health risk” or “Quitting smoking early is good for your health” is required to be printed on all the packs. A study showed that they were least effective in informing public of dangers of smoking, comparing to warning labels adopted by some other countries with more specific smoking hazards [21]. To educate the public with more specific health hazard of SHS is also very important to facilitate smoking bans in restaurants and bars, as shown by the regression analysis that those who knew more about SHS hazards were more likely to support governmental smoking bans in these venues and more willing to restrict or ban smoking in their own venues.

Though there were some knowledge gaps about SHS and its health effects, there was support for smoke-free environments in public places. Subjects might have over-reported their support for smoking regulations in hospitals, schools, public vehicles and offices due to social desirability or some other reasons, but this alone seems unlikely to account for the level of support reported. As for regulations in restaurants and bars, the tendency to over-report was probably stronger because owners were invited to the study by local CDCs or health institutes. However, all subjects were reminded at the beginning of invitations and interviews that smoking prohibition or restriction was not required in hospitality venues and the survey had nothing to do with any form of hygiene inspection. We would expect those owners and managers who opposed to prohibit or restricting smoking to report their opposition due to concerns that support reported by them might facilitate the implementation of smoking bans.

Supportive attitudes towards banning smoking in restaurants and bars differed by city. This might be attributed to different scopes of tobacco control initiatives in different cities. Beijing for example was the leading city in tobacco control and had initiated a series of activities on promoting smoke-free environments, while Wuhan had fewer government initiatives. Consistent with a review on public attitudes towards smoke-free policies by International Agency for Research on Cancer (IARC), this study found that smokers were less supportive of prohibiting or restricting smoking in restaurants and bars and the support for smoke-free bars was lower than for smoke-free restaurants [15].

There were several misunderstandings among hospitality owners and managers concerning smoking in restaurants and bars. First, many overestimated the impact of restricting smoking to designated sections on preventing exposure to SHS. This may partially explain the higher support for smoking restriction than for prohibition. Second, they believed that indoor air quality depended on ventilation more than prohibiting smoking. In fact, ventilation and any other engineering approaches cannot eliminate adverse health effects from exposure to SHS [22]. Lastly, most of them worried that smoking bans would reduce their revenues, a common belief that has been demonstrated to be false in a systematic review of the economic impacts of smoke-free laws in North America, Australia, and other Western countries [23]. To facilitate smoking bans in restaurants and bars, we should replace common misunderstanding with empirical evidence from China as well experiences from international practices.

The study found that owners who were unwilling to prohibit or restrict smoking in their own venues thought it was not their duty and it would decrease their revenues, and that owners from Chinese food restaurants and bars were least likely to voluntarily prohibit or restrict smoking in their own venues. All these indicate big challenges to depend on voluntary smoking restriction to reduce or eliminate SHS exposure in restaurants and bars in China. Without governmental regulations, which would set consistent smoking policies for all the hospitality venues and impact their revenues indifferently, owners were less likely to prohibit or restrict smoking voluntarily due to the concern that self-imposed smoking prohibition or restriction would bring negative impact on their revenues under the existing social context, that is, more than half (53%) of men smoking [10], and sharing cigarettes, “among men in particular” “is used as a way to break down social barriers, foster friendship, and promote bonding” [24].

## 5. Study Limitations and Strengths

The response rate of this study was 100%, probably because subjects were invited to the study through local CDCs or health institutes. As discussed previously, this might lead to over-reporting of existing smoking restrictions and support for banning smoking in restaurants and bars. It would be better to use an independent third party to conduct all the interviews to decrease these potential over-reports, however, this approach is often very expensive, and it cannot always be accommodated in a broad scale public health research like this study in China, and using the existing public health system is necessary. Furthermore, all subjects were assured that information obtained from interviews would not be linked with any hygiene inspection, and we would more like to regard this high response rate as strength rather than a limitation of the study.

The study focused specifically on restaurant and bar owners’ knowledge and attitudes regarding banning smoking in their own venues across different cities in the country, and it is able to provide key information for tobacco control initiatives in restaurants and bars in China and in other international countries. However, the five cities included in this study are not representative of all cities in China, and subjects interviewed were not from a random sample of hospitality venues. We are unable to estimate how the convenience sample would bias the results. Thus, any interpretation relating the study results to all restaurants and bars in any of or all the five cities or in China should be cautious.

Another limitation of the study is that for some restaurants and bars, especially those big ones, their managers other than their owners were interviewed, and no information was collected on whether a questionnaire was answered by an owner or a manager. Though the managers contacted the owners to get authorization to participate in the survey, their answers to the questionnaires might be quite different from the owners. And managers might not have the authority to decide to prohibit or restrict smoking voluntarily in their venues. However, managers’ support and willingness to do so may indicate likely compliance of any smoking prohibition or restriction in their venues.

Furthermore, there was potential social desirability bias, which might lead to over-reporting of knowledge on SHS hazards, existing voluntary smoking restrictions and support for smoking bans, and we were not able to estimate the scope of this bias. However, if findings from this study were attributed to social desirability bias to a great extent, it indicates that smoke-free hospitality venues are favored by the society to a noticeable extent; and if the social desirability was not significant to bias the findings, then the owners’ support presented significant evidence of feasibility to impose governmental restrictions in hospitality venues. In either case, it indicated the necessity of facilitating smoking bans in hospitality venues in China in aspect of social readiness.

## 6. Conclusions

Though restaurant and bar owners and managers have limited knowledge on specific health hazards of SHS, many of them support government regulation of smoking in public places. Fewer, however, are willing to prohibit or restrict smoking in their own establishment. Governmental regulation of smoking in hospitality venues is necessary to reduce health problems related to secondhand smoke in China. To garner adequate support for these policies at the national level, it may be necessary to complement international studies with China-specific evidence that smoking bans will not reduce hospitality revenues. It is also important to educate people so they understand that, even with better ventilation in restaurants and bars, and with smoking restricted to designated areas, substantial health risks from secondhand smoke remain, and include not only lung cancer but also asthma and heart disease.

## Figures and Tables

**Table 1.** Type of establishments and characteristics of restaurant and bar owners and managers interviewed, five Chinese cities, 2007.

	**Total n (%)**	**Beijing n (%)**	**Wuhan n (%)**	**Xi’an n (%)**	**Kunming n (%)**	**Guiyang n (%)**	**χ^2^test*p*value**
*Total*	814 (100.0)	161 (100.0)	172 (100.0)	161 (100.0)	160 (100.0)	160 (100.0)	
*Type of Establishment*							
Chinese dining	506 (62.2)	95 (59.0)	112 (65.1)	100 (62.1)	100 (62.5)	99 (61.9)	0.996
Chinese fast food	58 (7.1)	17 (10.6)	10 (5.8)	10 (6.2)	10 (6.3)	11 (6.9)	
Western dining	52 (6.4)	11 (6.8)	10 (5.8)	10 (6.2)	10 (6.3)	11 (6.9)	
Western fast food	49 (6.0)	11 (6.8)	10 (5.8)	10 (6.2)	9 (5.6)	9 (5.6)	
Bar	149 (18.3)	27 (16.8)	30 (17.4)	31 (19.3)	31 (19.4)	30 (18.8)	
*Gender*							
Male	413 (50.7)	93 (57.8)	71 (41.3)	84 (52.2)	82 (51.3)	83 (51.9)	0.049
Female	399 (49.0)	67 (41.6)	100 (58.1)	77 (47.8)	78 (48.8)	77 (48.1)	
Missing	2 (0.2)	1 (0.6)	1 (0.6)	0 (0.0)	0 (0.0)	0 (0.0)	
*Age*							
≤35	571 (70.0)	111 (68.9)	116 (67.4)	109 (67.7)	117 (73.1)	118 (73.8)	0.692
>35	239 (29.5)	50 (31.1)	54 (31.4)	50 (31.1)	43 (26.9)	42 (26.3)	
Missing	4 (0.5)	0 (0.0)	2 (1.2)	2 (1.2)	0 (0.0)	0 (0.0)	
*Education*							
≤High school	444 (54.5)	85 (52.8)	100 (58.1)	91 (56.5)	98 (61.3)	70 (43.8)	0.016
>High School	368 (45.2)	76 (47.2)	70 (40.7)	70 (43.5)	62 (38.8)	90 (56.3)	
Missing	2 (0.2)	0 (0.0)	2 (1.2)	0 (0.0)	0 (0.0)	0 (0.0)	
*Current Smoking*							
No	525 (64.5)	106 (65.8)	121 (70.3)	99 (61.5)	100 (62.5)	99 (61.9)	0.338
Yes	287 (35.3)	54 (33.5)	50 (29.1)	62 (38.5)	60 (37.5)	61 (38.1)	
Missing	2 (0.2)	1 (0.6)	1 (0.6)	0 (0.0)	0 (0.0)	0 (0.0)	

Note: results presented in this table represent simple aggregation of the survey results, and no sampling weighting was used.

**Table 2.** Reported SHS Exposure at work in the past week by workplace smoking policy for current nonsmoking restaurant and bar owners in five Chinese cities, 2007.

**Days Exposed**	**Restaurants Smoking policy at work**				**Bars Smoking policy at work**			
	**Prohibit % (n)**	**Restrict % (n)**	**Allow % (n)**	**Total % (n)**	**Prohibit % (n)**	**Restrict % (n)**	**Allow% (n)**	**Total % (n)**
None	51.1 (24)	14.4 (16)	6.1 (13)	14.3 (53)	0.0 (0)	17.6 (3)	7.7 (2)	10.2 (5)
1–2	21.3 (10)	15.3 (17)	9.0 (19)	12.4 (46)	33.3 (2)	17.6 (3)	11.5 (3)	16.3 (8)
3–4	10.6 (5)	18.0 (20)	9.9 (21)	12.4 (46)	0.0 (0)	17.6 (3)	3.8 (1)	8.2 (4)
5–7	17.0 (8)	52.3 (58)	75.0 (159)	60.8 (225)	66.7 (4)	47.1 (8)	76.9 (20)	65.3 (32)
Total	100.0 (47)	100.0 (111)	100.0 (212)	100.0 (370)	100.0 (6)	100.0 (17)	100.0 (26)	100.0 (49)

Notes: Fifty current nonsmokers answered “Don’t know” and 53 current nonsmokers did not answer to the question about how many days they were exposed to SHS at work, and three others answered “Don’t know” to the question about the smoking policy at work, thus a total of 419 (370 + 49) rather than 525 current nonsmokers are included in this study. Results presented in this table represent simple aggregation of the survey results, and no sampling weighting was used.

**Table 3.** Smoking policy favored for public places by restaurant and bar owners, five Chinese cities, 2007.

	**Smokers (%)**			**Non-smokers (%)**			**All subjects (%)**		
	**Prohibit**	**Restrict**	**Total**	**Prohibit**	**Restrict**	**Total**	**Prohibit**	**Restrict**	**Total**
School	84.3	15.0	99.3	87.6	11.8	99.4	86.4	13.0	99.4
Public vehicle	87.4	10.1	97.5	87.4	11.2	98.6	87.4	10.8	98.2
Hospital	69.6	30.4	100	74.7	24.8	99.4	73.3	26.3	99.6
Office	49.3	45.1	94.4	62.7	32.8	95.5	58.1	36.9	95.0
Restaurant	10.8	64.7	75.5	20.2	63.2	83.4	16.8	63.8	80.6
Bar	6.3	46.9	53.2	14.1	52.7	66.8	11.4	50.3	61.7

Notes: In mainland China, smoking is nationally prohibited in public vehicles, and classrooms, activity rooms and dorms for minors and smoking is usually restricted in some areas of hospitals. By the time of this study, smoking in offices and restaurants and bars is not regulated in the five cities; Results presented in this table represent simple aggregation of the survey results, and no sampling weighting was used.

**Table 4.** Owner’s and manager’s attitudes towards banning smoking in restaurants and bars, five Chinese cities, 2007.

	**total**	**What kind of smoking policy do you think restaurants/bars should have?^a^n (%)**						**Willing to do so^b^n (%)**
		**restaurants**			**bars**			
		**prohibit**	**restrict**	**allow**	**prohibit**	**restrict**	**allow**	
*Total*	814	137 (16.8)	519 (63.7)	106 (13.0)	92 (11.3)	411 (50.6)	195 (24.0)	433 (53.3)
*City*								
Beijing	161	36 (22.4)	106 (65.8)	11 (6.8)	26 (16.3)	88 (55.0)	21 (13.1)	108 (67.9)
Wuhan	172	26 (15.2)	89 (52.0)	45 (26.3)	20 (11.7)	67 (39.2)	67 (39.2)	83 (48.3)
Xi’an	161	36 (22.4)	100 (62.1)	11 (6.8)	18 (11.2)	78 (48.4)	32 (19.9)	76 (47.2)
Kunming	160	18 (11.3)	107 (66.9)	26 (16.3)	11 (6.9)	81 (50.6)	51 (31.9)	76 (47.5)
Guiyang	160	21 (13.1)	117 (73.1)	13 (8.1)	17 (10.6)	97 (60.6)	24 (15.0)	90 (56.3)
*Type of Establishment*								
Chinese dining	506	87 (17.2)	312 (61.7)	71 (14)	63 (12.5)	252 (49.9)	110 (21.8)	270 (53.6)
Chinese fast food	58	15 (25.9)	37 (63.8)	4 (6.9)	10 (17.2)	31 (53.4)	12 (20.7)	32 (55.2)
Western dining	52	3 (5.9)	42 (82.4)	4 (7.8)	3 (5.9)	29 (56.9)	12 (23.5)	36 (69.2)
Western fast food	49	17 (34.7)	28 (57.1)	3 (6.1)	8 (16.3)	26 (53.1)	11 (22.4)	38 (77.6)
Bar	149	15 (10.1)	100 (67.1)	24 (16.1)	8 (5.4)	73 (49.0)	50 (33.6)	57 (38.3)
*Gender*								
Male	413	64 (15.5)	259 (62.9)	58 (14.1)	45 (10.9)	204 (49.5)	111 (26.9)	210 (51.0)
Female	399	73 (18.3)	259 (64.9)	47 (11.8)	47 (11.8)	206 (51.8)	83 (20.9)	223 (56.0)
*Age*								
≤35	571	84 (14.7)	385 (67.4)	66 (11.6)	59 (10.3)	306 (53.6)	127 (22.2)	266 (46.7)
>35	238	51 (21.4)	132 (55.5)	40 (16.8)	32 (13.5)	104 (43.9)	67 (28.3)	111 (46.4)
*Education*								
≤High school	444	80 (58.4)	274 (52.9)	30 (16.2)	22 (11.9)	79 (42.7)	58 (31.4)	96 (51.9)
>High school	367	57 (41.6)	244 (47.1)	37 (14.3)	37 (14.3)	134 (51.9)	55 (21.3)	139 (53.7)
*Current smoking*								
Yes	287	106 (20.2)	332 (63.2)	62 (11.8)	74 (14.1)	276 (52.7)	99 (18.9)	125 (43.6)
No	525	31 (10.8)	185 (64.7)	44 (15.4)	18 (6.3)	134 (46.9)	95 (33.2)	308 (58.9)
*Knowledge scores*								
0–3	221	29 (13.1)	132 (59.7)	38 (17.2)	15 (6.8)	98 (44.3)	60 (27.1)	101 (45.7)
4–6	592	108 (18.2)	387 (65.4)	68 (11.5)	77 (13.0)	313 (53.0)	135 (22.8)	332 (56.1)
*Banning smoking would not reduce revenues*								
Disagree/DNK ^c^	447	42 (9.4)	295 (66.0)	72 (16.1)	34 (7.6)	219 (49.0)	121 (27.1)	194 (43.4)
Agree	365	95 (26.0)	223 (61.1)	34 (9.3)	58 (15.9)	191 (52.5)	74 (20.3)	238 (65.6)

Note: results in presented in this table represent simple aggregation of the survey results, and no sampling weighting was used.

a Answers to this question included “prohibit”, “restrict” “allow” and “ I don’t care”; only the first three options are presented in this table;

b The question was that “Are you willing to prohibit or restrict smoking in your own restaurant or bar?” Answers to this question included “yes”, “no” and “refuse to answer”; only results of the first option are presented in this table;

c Disagree or don’t know.

**Table 5.** Odds Ratios for owners’ attitudes towards prohibiting or restricting smoking in restaurants and bars, five Chinese cities, 2007.

	**Support prohibiting or restricting smoking in restaurants OR (95% CI)^a^**	**Support prohibiting or restricting smoking in bars OR (95% CI)^a^**	**Willing to prohibit or restrict smoking in their own venues OR (95% CI)^a^**
*City*			
Beijing	(reference)	(reference)	(reference)
Wuhan	0.3 (0.1, 0.5) ^***^	0.4 (0.3, 0.6) ^***^	0.4 (0.3, 0.7) ^***^
Xi’an	0.8 (0.4, 1.5)	0.6 (0.4, 0.99) ^**^	0.4 (0.3, 0.7) ^***^
Kunming	0.6 (0.3, 1.0)	0.6 (0.4, 0.9) ^**^	0.5 (0.3, 0.7) ^***^
Guiyang	1.0 (0.5, 2.0)	1.1 (0.7, 1.8)	0.7 (0.4, 1.1)
*Type of Establishment*			
Chinese dining	(reference)	—	(reference)
Chinese fast food	2.3 (0.9, 5.6)	—	0.9 (0.5, 1.7)
Western dining	1.7 (0.7, 4.2)	—	1.9 (1.01, 3.7) ^*^
Western fast food	2.5 (0.8, 7.5)	—	2.6 (1.3, 5.3) ^**^
Bar	1.0 (0.6, 1.6)	—	0.6 (0.4, 0.9) ^**^
*Age*			
≤35	(reference)	(reference)	—
>35	0.7 (0.5, 1.1)	0.8 (0.6, 1.1)	—
*Current smoking*			
No	(reference)	(reference)	(reference)
Yes	0.6 (0.4, 0.9) ^**^	0.6 (0.4, 0.8) ^***^	0.6 (0.4, 0.8) ^***^
*Knowledge scores*			
0–3	(reference)	(reference)	(reference)
4–6	1.9 (1.3, 2.9) ^***^	1.9 (1.3, 2.6) ^***^	1.4 (1.01, 2.0) ^*^
*Banning smoking would not reduce revenues*			
Disagree/DNK ^b^	(reference)	(reference)	(reference)
Agree	2.2 (1.5, 3.2) ^***^	1.6 (1.2, 2.2) ^***^	2.3 (1.7, 3.1) ^***^

Notes: the three columns presenting ORs are results from separate logistic regression models which simultaneously adjust for the covariates illustrated on Table 4; variables not included in this table or labeled with “—” were excluded because their *p* values from Wald test were greater than 0. 20; Results presented in this table represent simple aggregation of the survey results, and no sampling weighting was used.

a OR: Odds Ratio; CI: Confidence Interval;

b Disagree or don’t know;

* *p*<0.05,

** *p*<0.01,

*** *p*<0.001.

## References

[1] Cal/EPA (2005). Proposed Identification of Environmental Tobacco Smoke as a Toxic Air Contaminant.

[2] IARC (2004). Monographs on the Evaluation of Carcinogenic Risks to Humans. Tobacco Smoke and Involuntary Smoking, Volume 83.

[3] US Department of Health and Human Services (USDHHS) (2006). The Health Consequences of Involuntary Exposure to Tobacco Smoke: A Report of the Surgeon General.

[4] Lightwood JM, Glantz SA (2009). Declines in acute myocardial infarction after smoke-free laws and individual risk attributable to secondhand smoke. Circulation.

[5] Meyers DG, Neuberger JS, He J (2009). Cardiovascular effect of bans on smoking in public places. J. Amer. Coll. Cardiol.

[6] Ong MK, Glantz SA (2004). Cardiovascular health and economic effects of smoke-free workplaces. Amer. J. Med.

[7] Sargent RP, Shepard RM, Glantz SA (2004). Reduced incidence of admissions for myocardial infarction associated with public smoking ban: Before and after study. Brit. Med. J.

[8] American Nonsmokers’ Rights Foundation (ANRF) (2010). Summary of 100% Smokefree State Laws and Population Protected by 100% U.S. Smokefree Laws.

[9] WHO (2009). World Health Organization Report on the Global Tobacco Epidemic, 2009: Implementing Smoke-Free Environments.

[10] CDC (2010). Smoking and Tobacco Use: GATS: Fact Sheet: China: 2010.

[11] Li Y, Jiang Y, Yang Y, Nan Y, Feng G, Zhou G, Li M, Zhang C (2007). Current situation of law and regulation for banning smoking in public places in China. J. Environ. Health.

[12] Ministry of Health (2007). China Tobacco Control Report: 2007.

[13] Beijing Government (2008). Regulations on Smoking in Public Places in Beijing, Rule 204.

[14] Yang G, Ma J, Liu N, Zhou L (2005). Smoking and passive smoking in China, 2002. Chin. J. Epidemiol.

[15] IARC (2009). Handbooks of Cancer Prevention, Tobacco Control, Vol. 13: Evaluating the Effectiveness of Smoke-Free Policies.

[16] National Bureau of Statistics of China (2007). China Statistic Year Book 2007.

[17] Liu R, Yang Y, Travers M, Fong GT, O’Connor RJ, Hyland A, Li L, Nan Y, Feng G, Li Q (2010). A cross-sectional study on levels of second-hand smoke in restaurants and bars in five cities in China. Tob. Control.

[18] Yang Y, Jiang Y, Wu X, Feng G (2008). Analysis of policies on banning smoking in public places and their implementation in China. Chin. J. Health Edu.

[19] National Bureau of Statistics of China (2006). China Labour Statistical Yearbook 2006.

[20] Liu R, Yang Y, Liu X, Chang A, Gong J, Zhao B, Liu T, Jiang Y, Hyland A, Li Q (2008). Knowledge and attitudes towards secondhand smoke among hospitality patronage in five cities in China. Chin. J. Epidemiol.

[21] Fong GT, Hammond D, Jiang YA, Li QA, Quah ACK, Driezen P, Yan M (2010). Perceptions of tobacco health warnings in China compared with picture and text-only health warnings from other countries: An experimental study. Tob. Control.

[22] ASHRAE (2008). ASHRAE Position Document on Environmental Tobacco Smoke.

[23] Hyland A, Vena C, Cummings MK (2000). A review of the economic effect of smoke-free restaurant and bar policies on the hospitality economy. Epidemiology.

[24] Ma SJ, Hoang MA, Samet JM, Wang JF, Mei CZ, Xu XF, Stillman FA (2008). Myths and attitudes that sustain smoking in China. J. Health Commun.

